# Bioanalysis of MMR and KRAS – a key factor in diagnosis of colorectal cancer

**DOI:** 10.1039/d3ra04260j

**Published:** 2023-08-11

**Authors:** Raluca-Ioana Stefan-van Staden, Alexandru Adrian Bratei, Ruxandra-Maria Ilie-Mihai, Damaris-Cristina Gheorghe, Bianca Maria Tuchiu, Simona Gurzu

**Affiliations:** a Laboratory of Electrochemistry and PATLAB, National Institute of Research for Electrochemistry and Condensed Matter 202 Splaiul Independentei Str. 060021 Bucharest-6 Romania ralucavanstaden@gmail.com +40213163113 +40751507779; b Faculty of Chemical Engineering and Biotechnologies, Politehnica University of Bucharest Bucharest Romania; c Department of Pathology, George Emil Palade University of Medicine, Pharmacy, Sciences and Technology Targu-Mures Romania

## Abstract

Two miniaturized electrochemical devices were designed for the simultaneous bioanalysis of MMR (MLH1, MSH2, MSH6, PMS2), and of KRAS in whole blood, urine, saliva, and tumoral tissues. The devices comprised besides the electronic part of the potentiostat a combined 3D stochastic microsensor (combined microplatform) with the sensing part based on the modification of graphene decorated with nitrogen, sulfur and boron (NSB-EGR) modified with two types of frutafit: FTEX and FHD. For the assay of MSH2, MSH6, KRAS, and PMS2 higher sensitivities were recorded when the microdevice based on FHD was used, while for the assay of MLH1 the best sensitivity was achieved by using the microdevice based on FTEX. While the limits of quantification for MLH1, MSH2, and PMS2 were not influenced by the modifier, the microdevice based on FHD provided the lowest limit of quantification for KRAS, the microdevice based on FTEX provided the lowest limit of quantification for MSH6. The validation tests performed proved that recoveries of MLH1, MSH2, MSH6, PMS2, and of KRAS in whole blood, urine, saliva, and tumoral tissues higher than 98.50% with RSD (%) values lower than 0.10% were recorded.

## Abstract 2

Bioanalysis of MLH1, MSH2, MSH6, PMS2, and KRAS using combined microplatforms based on graphenes and inulins proved to be a key factor in diagnosis of colorectal cancer.

## Graphical abstract

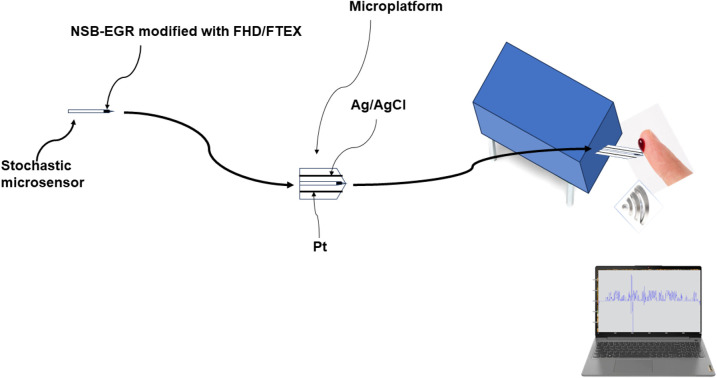

## Introduction

Due to the frequency and incidence of colorectal cancer (CRC), molecular classification and pathology mechanisms have been intensively studied during recent years.^1^ The main mechanism for the molecular pathogenic process related to CRC development follows the microsatellite instability (MSI) pathway.^2,3^ The identification of MSI colorectal cancers, especially the high instability (MSI-H) ones, is very important as MSI is a common feature of Lynch syndrome^4–6^ and MSI-H colorectal cancers can be non-responsive to 5-fluorouracil chemotherapy,^7^ therefore they are now tested for a potential treatment with newer immunotherapies.^8,9^

MSI status is due to a defective mismatch repair (dMMR) which mostly occurs due to mutations in the MLH1, MSH2, MSH6 and PMS2 genes. Failure in the MMR system function leads to the accumulation of errors within the genome and therefore to tumorigenesis. Another protein related to cancer development is KRAS, which is a GTPase transductor protein responsible for the regulation of cellular growth and differentiation.^10^ Mutations in the KRAS gene could lead to a continuous activation of KRAS pathway and thus, to cancer development.

Current guidelines recommend dMMR screening for all colorectal cancer patients to identify a potential Lynch syndrome and the patients to benefit from further counseling and genetic testing.^11–15^ The screening can be done by using immunohistochemistry to evaluate the loss of protein expression^16,17^ or MSI testing to evaluate unstable microsatellite regions resulting from dMMR.^18–20^ KRAS also has a very important role in colorectal cancer.^21^

This paper proposed two miniaturized electrochemical devices for simultaneous assay of MMR (MLH1, MSH2, MSH6, PMS2), and KRAS in whole blood, saliva, urine, and tumoral tissues. The novelty is given by the design of the electrochemical devices used for the fast simultaneous screening tests of biological samples, and by the design of the 3D combined stochastic microsensors, by utilizing a 3D printer to produce the support of the stochastic microsensor, reference sensor, and of the auxiliary sensor; moreover, the composition of the paste (the active side of the stochastic microsensor) is new – the graphene decorated with nitrogen, boron, and sulfur being modified with two types of frutafit: FHD, and FTEX.

The stochastic mode used for all measurements in this paper is based on the channel conductivity.^22–24^ There is a two-step process: qualitative step – when the MMR and KRAS are recognized based on their signatures (the process taking place is: the molecules enter one by one into the channel, bocking it, and the current drops to zero value – the time spent at this value is the one needed for the molecule to get inside the channel, and therefore it is called the signature of the molecule), and a second step on which the molecule inside the channel is undergoing binding and redox processes (the qualitative step, characterized through the measured *t*_on_ value – the time needed for the molecule to change its sign during the redox process). The advantages of using the stochastic mode *versus* other electrochemical methods are the following: the sample does not need any processing before the measurements; the complexity of the matrix does not influence the results of the measurement; the signature is associated with a high reliable qualitative analysis being dependent only on the size, geometry, and velocity of the molecule.

## Experimental

### Materials and reagents

Frutafit HD and frutafit TEX were purchased from Sensus (Roosendaal, The Netherlands). MLH1, MSH2, MSH6, PMS2, and KRAS were purchased from Sigma Aldrich (Milwaukee, USA); the paraffin oil was purchased from Fluka (Buchs, Switzerland). Monosodium phosphate and disodium phosphate were used for the preparation of phosphate buffer, pH = 7.5. Deionized water obtained from a Millipore Direct-Q 3 System was used for the preparation of all solutions from 10^−22^ to 10^−2^ g mL^−1^ magnitude order. Nitrogen (9.3%) and boron (2.4%) – dopped graphene (NB-DG) was provided by the National Institute of Research and Development of Isotopic and Molecular Technologies, Cluj-Napoca, Romania within the grant of the Ministry of Research, Innovation and Digitization, CNCS/CCCDI – UEFISCDI, project number PN-III-P4-ID-PCCF-2016-0006.

### Apparatus and methods

A microdevice EmSTAT Pico (PalmSens, Houten, The Netherlands) linked to a personal smartphone (PsTrace 5.8 software), through a USB connection was used for all measurements. The combined stochastic microsensor was integrated into a microplatform containing also the reference (Ag/AgCl) microsensor as well as the auxiliary (Pt wire) microsensor.

### Design of the combined microplatforms

#### Design of the stochastic microsensors

50 μL of FHD and FTEX (10^−3^ mol L^−1^), respectively, were each added to 50 mg dopped graphene (NSB-EGR) paste (made by mixing NSB-EGR powder with paraffin oil). Each of the pastes were placed in 3D printed minitubes with internal diameter of 20 μm; an Ag wire made the connection between the paste and the external circuit.

**Scheme 1:**
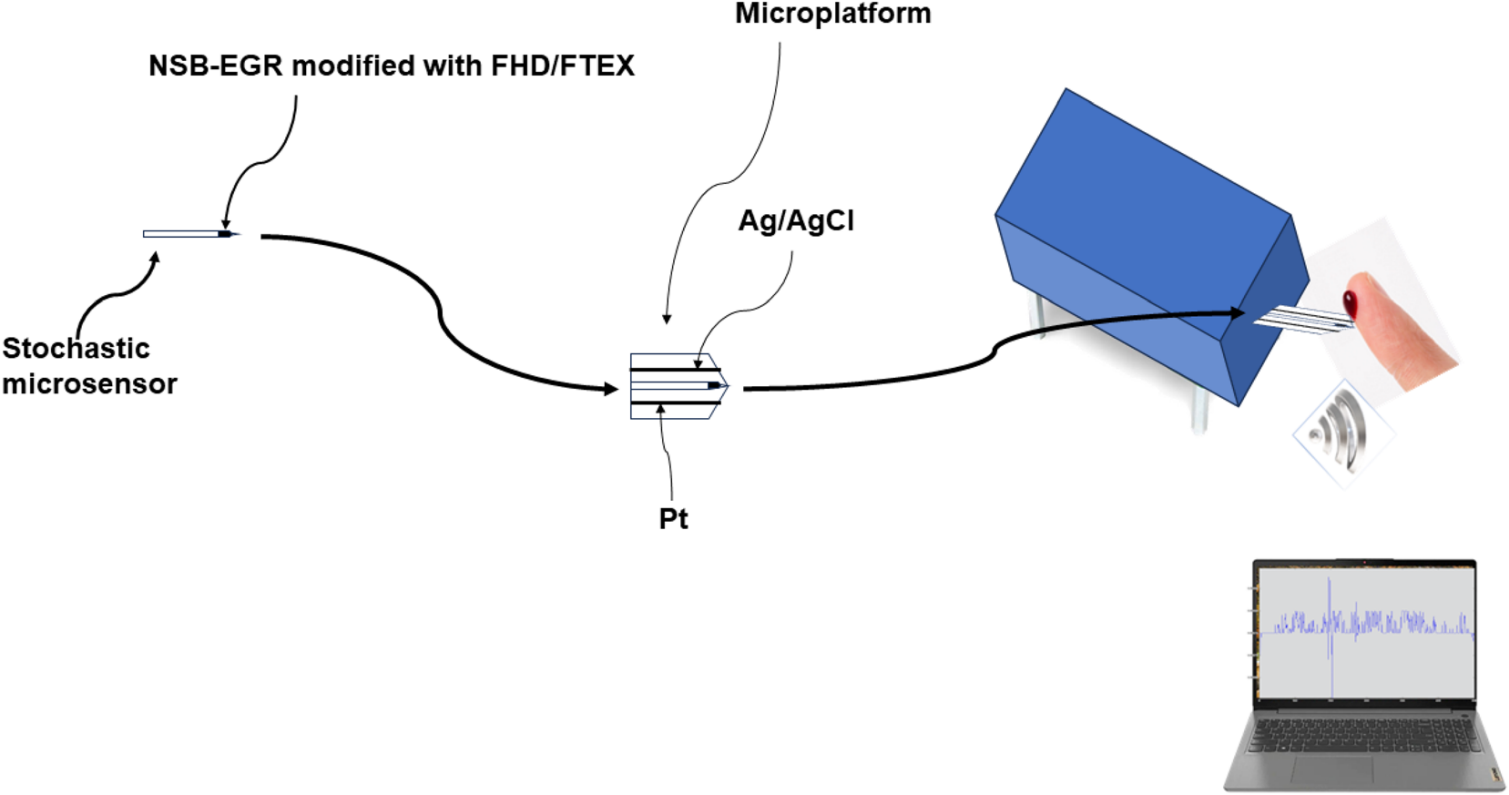
Design of the stochastic microsensor, and microplatform of measurement used in simultaneous assay of MLH1, MSH2, MSH6, PMS2, and of KRAS in whole blood, urine, saliva, and tumoral tissues.

The stochastic microsensor was integrated in a microplatform together with the counter electrode (platinum wire), and the reference electrode (Ag/AgCl electrode) (Scheme 1).

Before and after each measurement, cleaning with deionized water and soft drying with an adsorbant paper were performed. When not in use, the microplatforms were kept in a dry place, at room temperature.

### Stochastic method

**Fig. 1:**
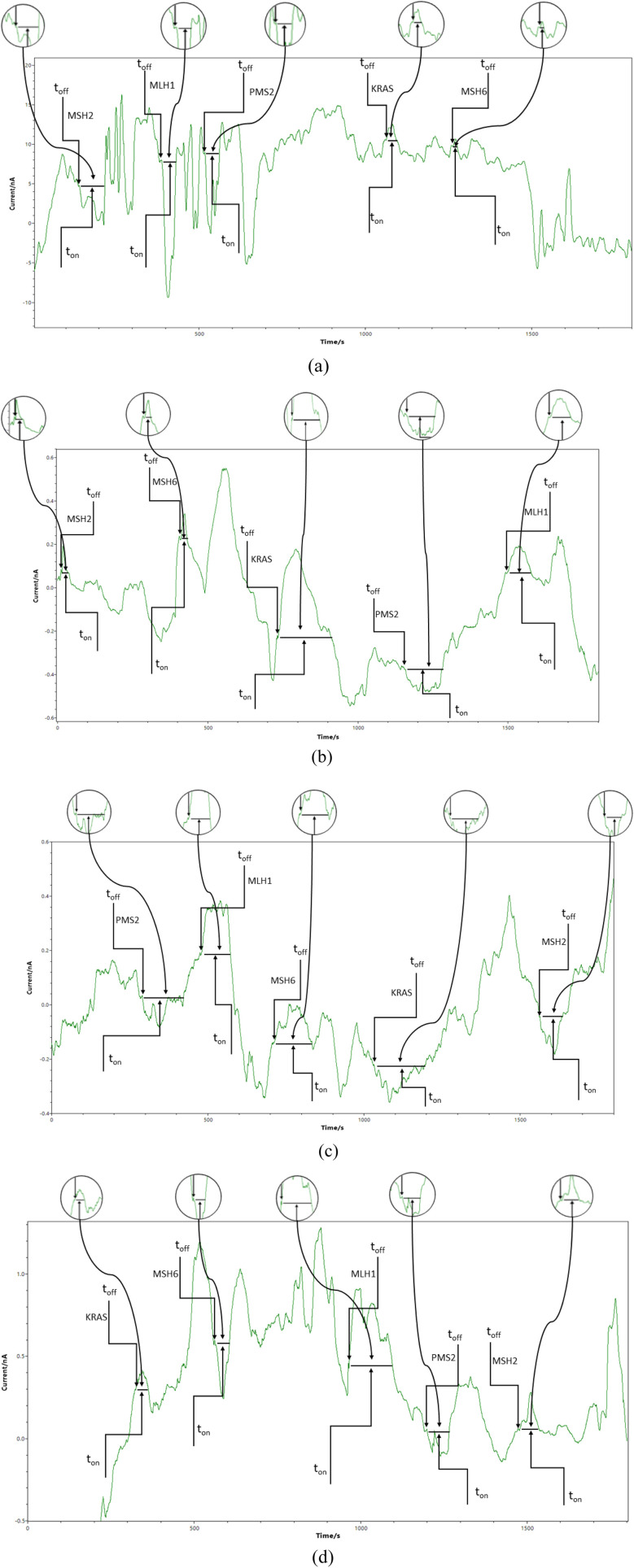
Typical diagrams obtained by screening (a) whole blood, (b) saliva, (c) urine, and (d) tumoral tissues with the microplatform based on FHD/NSB-EGR.

**Fig. 2:**
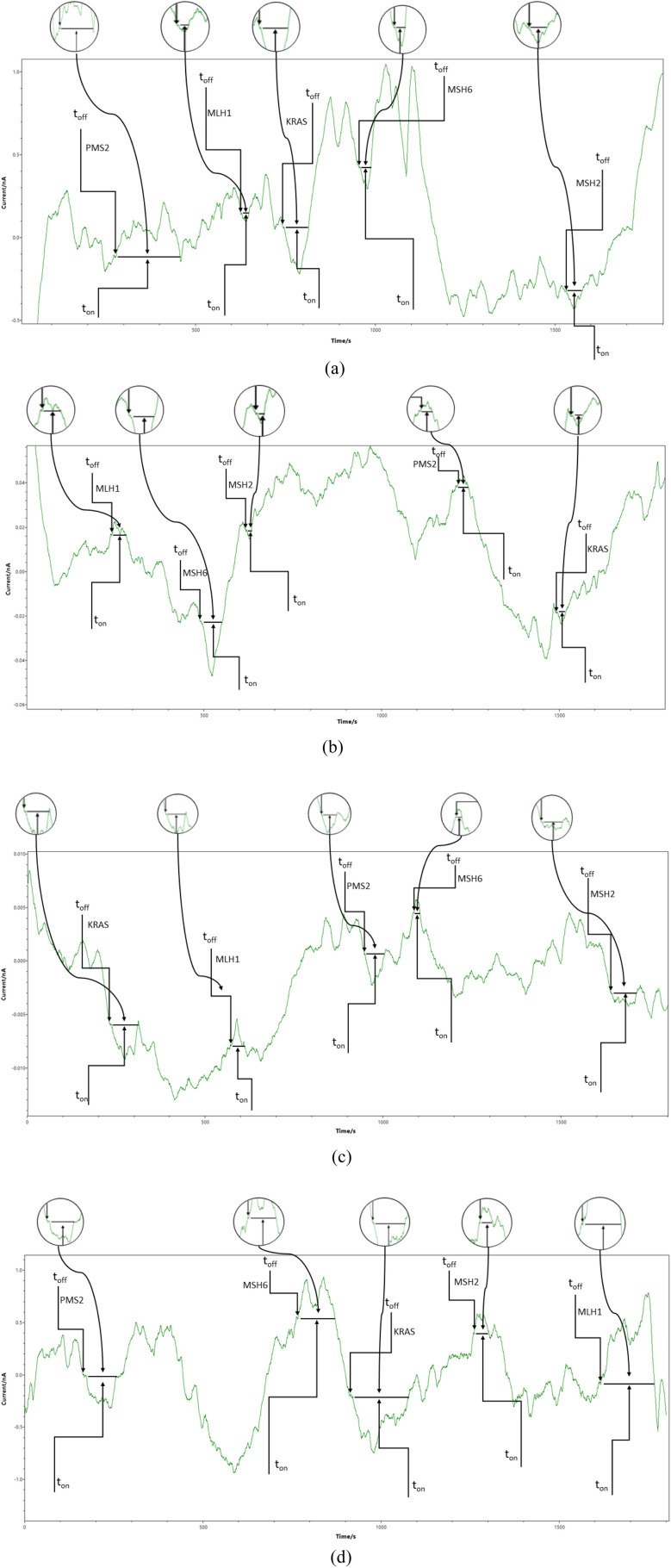
Typical diagrams obtained by screening (a) whole blood, (b) saliva, (c) urine, and (d) tumoral tissues with the microplatform based on FTEX/NSB-EGR.

Chronoamperometry was used for all measurements. A potential of 125 mV *vs.* Ag/AgCl was applied, and diagrams were recorded (Fig. 1 and 2). The signatures (t_off_ values) of the MLH1, MSH2, MSH6, PMS2, and of KRAS, were identified in the diagrams, and served as recognition elements for the biomarkers. The values of *t*_on_ served for all quantitative measurements. Series of solutions of MLH1, MSH2, MSH6, PMS2, and of KRAS (with various concentrations) were used for the calibration of the microplatforms. The equations of calibration obtained for the biomarkers using each of the two microplatforms were based on the determination of the *t*_on_ value (read in between two consecutive *t*_off_ values); *a*, and *b* parameters from the equation of calibration 1/*t*_on_ = *a* + *b* × Conc._biomarker_ were determined using the linear regression method. For the screening of whole blood, urine, saliva, and tumoral tissue, the biomarkers were recognized based on their signature (*t*_off_ values) (Fig. 1 and 2), the *t*_on_ values were read and inserted into the equation of calibration for the determination of the concentration of MLH1, MSH2, MSH6, PMS2, and of KRAS in whole blood, urine, saliva, and tumoral tissue.

## Samples

Over 300 samples of whole blood, tumoral tissue, saliva, and urine samples were collected from the patients confirmed with colon cancers, and used for the measurements, without any pretreatment before the analysis. None of the patients was undergoing any type of treatment for cancer at the collection of the samples. These samples were obtained from the Emergency Clinical Hospital of County Targu-Mures, which was granted permission to conduct the research by the Ethics Committee with the number 32647/14.12.2018, and from the Clinical Hospital County Targu-Mures, which was granted permission to conduct the research by the Ethics Committee with the number 3206/28.02.2019. Informed consent was obtained from all patients.

## Response characteristics of the combined microplatforms

Stochastic mode was applied to determine all response characteristics of the proposed combined microplatforms. Different signatures were obtained for MLH1, MSH2, MSH6, PMS2, and of KRAS, when the same microplatform was used, proving that a reliable molecular recognition can be performed (Table 1). Response characteristics of the proposed combined microplatforms like, sensitivity, linear concentration range, limit of determination, were determined for both combined microplatforms (Table 1). Lower limits of determination – of fg mL^−1^ were obtained using the combined microplatforms. For the assay of MLH1, the highest sensitivity was recorded when FTEX was used in the design of the combined microplatform, while the widest linear concentration range was recorded when the FHD based combined microplatform was used. For the assay of MSH2, the widest linear concentration range was recorded when the FTEX based combined microplatform was used, while the lowest limit of determination and the highest sensitivity was recorded when the FHD based combined microplatform was utilized for the assay of MSH2. The lowest limit of determination obtained for the assay of MSH6, as well as the widest linear concentration range, and the highest sensitivity were reported for the combined microplatform based on FTEX. The widest linear concentration range and the highest sensitivity for the assay of PMS2 were obtained when the combined microplatform based on FHD was used. For the assay of KRAS, the widest linear concentration range was recorded when the combined microplatform based on FHD was used, and the highest sensitivity was obtained when the combined microplatform based on FTEX was used.

**Table 1:** Response characteristics of the miniplatforms used for the assay of MLH1, MSH2, MSH6, PMS2, and of KRAS

Combined microplatform based on NSB-EGR and	Signature, *t*_off_ (s)	Linear concentration range (g mL^−1^)		Calibration equations; the correlation coefficient, *r*^a^	Sensitivity (s^−1^ μg^−1^ mL)	LOQ (fg mL^−1^)
**MLH1**						
FHD	1.2		3.20 × 10^−16^–3.20 × 10^−5^	1/*t*_on_ = 0.11 + 2.06 × 10^−2^ × *C*; *r* = 0.9995	2.06 × 10^−2^	0.32
FTEX	2.1		3.20 × 10^−15^–3.20 × 10^−6^	1/*t*_on_ = 0.05 + 1.03 × 10^−1^ × *C*; *r* = 0.9902	1.03 × 10^−1^	3.20

**MSH2**						
FHD	2.0		1.00 × 10^−15^–1.00 × 10^−9^	1/*t*_on_ = 0.06 + 2.33 × 10^2^ × *C*; *r* = 0.9994	2.33 × 10^2^	1.00
FTEX	1.1		1.00 × 10^−14^–1.00 × 10^−5^	1/*t*_on_ = 0.10 + 37.56 × *C*; *r* = 0.9979	37.56	10.00

**MSH6**						
FHD	1.8		2.30 × 10^−9^–2.30 × 10^−5^	1/*t*_on_ = 0.16 + 1.02 × 10^−2^ × *C*; *r* = 0.9947	1.02 × 10^−2^	2.30 × 10^6^
FTEX	3.4		2.30 × 10^−15^–2.30 × 10^−6^	1/*t*_on_ = 0.11 + 5.91 × 10^−3^ × *C*; *r* = 0.9907	5.91 × 10^−3^	2.30

**PMS2**						
FHD	1.4		2.70 × 10^−15^–2.70 × 10^−5^	1/*t*_on_ = 0.15 + 1.71 × 10^4^ × *C*; *r* = 0.9996	1.71 × 10^4^	2.70
FTEX	2.5		2.70 × 10^−15^–2.70 × 10^−6^	1/*t*_on_ = 0.09 + 2.00 × 10^−2^ × *C*; *r* = 0.9949	2.00 × 10^−2^	2.70

**KRAS**						
FHD	1.6		2.20 × 10^−15^–2.20 × 10^−5^	1/*t*_on_ = 0.06 + 9.50 × 10^−3^ × *C*; *r* = 0.9976	9.50 × 10^−3^	2.20
FTEX	1.3		2.20 × 10^−15^–2.20 × 10^−6^	1/*t*_on_ = 0.13 + 2.89 × 10^3^ × *C*; *r* = 0.9967	2.89 × 10^3^	2.20

a <*C* > – concentration = μg mL^−1^; <*t*_on_> = *s*; LOQ – limit of quantification.

Reproducibility and stability studies were performed for each type of combined microplatform. Ten combined microplatforms based on FHD, and on FTEX, respectively, were designed accordingly with the procedure described above. Measurements of the sensitivities were performed for each combined microplatform, and calculations of %, RSD were performed. Values for the %, RSD of the sensitivities calculated were less than 0.27% for the combined microplatform based on FHD while when FTEX was used %, RSD values less than 0.12% were recorded, proving the design’ reproducibility of combined microplatforms. The 20 combined microplatforms' sensitivities were further checked for 30 days in order to establish their stability in time; for all tested combined microplatforms, %, RSD values less than 0.51% were recorded during the 30 days. The variance recorded for measurements performed using both microplatforms when used for simultaneous assay of MLH1, MSH2, MSH6, PMS2, and of KRAS in whole blood, urine, saliva, and tissue samples, did not exceeded 0.10.

## Bioanalysis of MMR: MLH1, MSH2, MSH6, PMS2, and of KRAS, using the combined microplatforms

The proposed combined microplatforms were used for the bioanalysis of 300 samples of whole blood, saliva, urine, and tumoral tissues from patients confirmed with colorectal cancer. Diagrams were recorded (Fig. 1 and 2) and used for molecular recognition of MMR and KRAS based on their signatures (*t*_off_ values) as well. After the identification of each of MLH1, MSH2, MSH6, PMS2, and of KRAS, their concentration was determined accordingly with the procedure described in the Stochastic method paragraph.

**Fig. 3:**
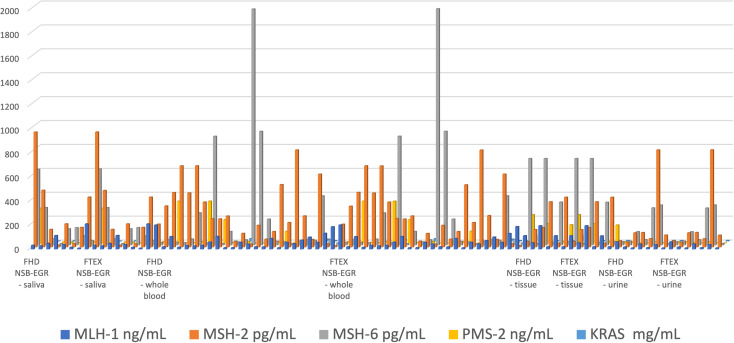
Determination of MLH1, MSH2, MSH6, PMS2, and KRAS in whole blood, saliva, urine, and tumoral tissue samples using the combined microplatforms based on FHD/NSB-EGR, and on FTEX/NSB-EGR.

A very good correlation between the results obtained using the combined microplatform based on FHD and using the combined microplatform based on FTEX (Fig. 3) were obtained for all samples: MLH1, MSH2, MSH6, PMS2, and KRAS in whole blood, saliva, urine, and tumoral tissue samples.

The %, RSD values associated to Fig. 3 are shown in Table 2. The values determined shown a high reproducibility of the measurements performed with the combined microplatform.

**Table 2:** The %, RSD average values recorded for the determination of MLH1, MSH2, MSH6, PMS2, and KRAS in biological samples

Combined microplatform based on NSB-EGR and		%, RSD									
		FHD NSB-EGR					FTEX NSB-EGR				
Biomarker		MLH-1	MSH-2	MSH-6	PMS-2	KRAS	MLH-1	MSH-2	MSH-6	PMS-2	KRAS
Biological fluid	Saliva	0.02	0.02	0.02	0.02	0.02	0.02	0.02	0.03	0.02	0.03
	Whole blood	0.02	0.03	0.02	0.02	0.02	0.02	0.03	0.02	0.02	0.02
	Tissue	0.02	0.02	0.02	0.03	0.03	0.02	0.02	0.03	0.02	0.02
	Urine	0.02	0.02	0.03	0.02	0.02	0.02	0.02	0.03	0.02	0.02

The paired Student's *t*-test was performed at 99.00% confidence level for all biomarkers: MLH1, MSH2, MSH6, PMS2, and KRAS. The calculated values for the *t*-test were lower than 3.21 (tabulated value at 99.00% confidence level is 4.13), proving that there is no significant difference between the results obtained using the two combined microplatforms based on FHD, and on FTEX.

Apart from the *t*-test, recovery tests of MLH1, MSH2, MSH6, PMS2, and KRAS were performed for whole blood, saliva, urine, and tumoral tissue samples. An initial screening was done to determine the amounts of MLH1, MSH2, MSH6, PMS2, and KRAS in whole blood, saliva, urine, and tumoral tissue samples. Ten different amounts of MLH1, MSH2, MSH6, PMS2, and KRAS were added to the real samples, and the final concentrations were determined. The added amounts of MLH1, MSH2, MSH6, PMS2, and KRAS in whole blood, saliva, urine, and tumoral tissue samples were compared with the found amounts. The results are given in Table 3.

**Table 3:** Recovery of MLH1, MSH2, MSH6, PMS2, and KRAS from whole blood, saliva, urine, and tumoral tissue samples (*N* = 10)

Combine microplatform based on NSB-EGR and		Recovery, %				
		MLH-1	MSH-2	MSH-6	PMS-2	KRAS
**Whole blood**						
FHD		99.99 ± 0.02	99.96 ± 0.01	99.83 ± 0.02	99.87 ± 0.02	99.95 ± 0.02
FTEX		99.95 ± 0.03	99.47 ± 0.01	99.91 ± 0.01	99.87 ± 0.03	99.96 ± 0.02

**Saliva**						
FHD		99.21 ± 0.03	99.21 ± 0.02	99.88 ± 0.01	99.12 ± 0.03	99.77 ± 0.04
FTEX		99.77 ± 0.05	99.30 ± 0.01	99.90 ± 0.02	95.43 ± 0.04	99.43 ± 0.02

**Urine**						
FHD		99.00 ± 0.02	99.20 ± 0.04	99.11 ± 0.02	99.12 ± 0.02	99.18 ± 0.04
FTEX		99.11 ± 0.04	99.22 ± 0.02	99.05 ± 0.01	99.08 ± 0.03	99.21 ± 0.02

**Tumoral tissue**						
FHD	98.90 ± 0.03		98.60 ± 0.03	98.77 ± 0.02	98.90 ± 0.03	98.73 ± 0.01
FTEX	99.00 ± 0.02		98.75 ± 0.04	98.97 ± 0.01	99.00 ± 0.02	98.78 ± 0.02

The performed recovery tests show high values for recoveries (all higher than 98.50%) with very low RSD (%), lower than 0.06%, when 10 measurements were performed. Accordingly, high accuracy and precision were achieved when the proposed combined microplatforms were used for the bioanalysis of the samples.

Compared to the results obtained for the assay of KRAS and MLH1, MSH2, MSH6, PMS2,^25,26^ using stochastic sensors, the working concentration ranges are wider, and the limits of determination are far lower, favorizing the identification and quantification of MLH1, MSH2, MSH6, PMS2, and KRAS in whole blood, saliva, urine, and tumoral tissue samples, at a very early stage of colon cancer.

## Conclusions

The miniplatforms based on 3D stochastic microsensors can be successfully used for the screening of whole blood, saliva, urine, and tissue samples for MLH1, MSH2, MSH6, PMS2, and KRAS. Accordingly, noninvasive screening tests based on the screening of urine and saliva can be performed, as well as a minim invasive screening test based on the screening of whole blood can be performed with a low cost, in less than 15 minutes. The miniplatforms may also be used during the surgeries for the screening tests of the tumoral tissues, being able to provide qualitative and quantitative information about the MLH1, MSH2, MSH6, PMS2, and KRAS, valuable for the medical doctor in taking immediate decision regarding the surgery, and the state of health of the patient.

## Ethical statement

The human biological samples were obtained from the Emergency Clinical Hospital of County Targu-Mures, which was granted permission to conduct the research by the Ethics Committee with the number 32647/14.12.2018, and from the Clinical Hospital County Targu-Mures, which was granted permission to conduct the research by the Ethics Committee with the number 3206/28.02.2019, accordingly with the European Commission Guidance document. Informed consent was obtained from all patients within the project PN-III-P4-ID-PCCF-2016-0006.

## Authors contributions

Methodology RISvS; coordination of project RISvS; experimental work: AAB, RMIM, DCG, BMT; validation: RISvS, AAB, SG; writing and correcting the MS: RISvS, AAB, RMIM, DCG, BMT.

## Conflicts of interest

There are no conflicts to declare.

## Supplementary Material
